# Early magnesium treatment after aneurysmal subarachnoid hemorrhage: an individual patient data meta-analysis

**DOI:** 10.1186/2197-425X-3-S1-A774

**Published:** 2015-10-01

**Authors:** WM van den Bergh, writing groups of MASH-I, IMASH, MASH-II, MASH and FAST-MAG

**Affiliations:** University Medical Center Groningen, Critical Care, Groningen, Netherlands

## Introduction

Delayed cerebral ischemia (DCI) is an important cause of poor outcome after aneurysmal subarachnoid hemorrhage (SAH). Trials on magnesium treatment starting < 4 days after symptom onset found no effect on poor outcome or DCI in SAH. Earlier instalment of treatment might be more effective, but individual trials had not enough power for such a sub-analysis.

## Objectives

We performed an individual patient data meta-analysis to study whether magnesium is effective when given within different time frames within 24 hours after the SAH.

## Methods

Patients were divided into categories according to the delay between symptom onset and start of the study medication: < 6 hours, 6-12 hours, 12-24 hours, > 24 hours. We calculated adjusted risk ratios (aRR) with corresponding 95% confidence intervals (CI) for magnesium versus placebo treatment for poor outcome and DCI.

## Results

We included 5 trials totalling 1981 patients, 83 patients started treatment < 6 hours. For poor outcome the aRRs of magnesium treatment for start < 6 hours were 1.44 (95%CI:0.83-2.51); for 6-12 hours 1.03(0.65-1.63), for 12-24 hours 0.84(0.65-1.09) and for >24 hours 1.06(0.87-1.31), and for DCI, < 6 hours 1.76(0.68-4.58), for 6-12 hours 2.09(0.99-4.39), for 12-24 hours 0.80(0.56-1.16) and for > 24 hours 1.08(0.88-1.32).

## Conclusions

This meta-analysis suggests no beneficial effect of magnesium treatment on poor outcome or DCI when started early after SAH onset. Although the number of patients was small and a beneficial effect cannot be definitively excluded, we found no justification for a new trial with early magnesium treatment after SAH.

**Figure 1:**
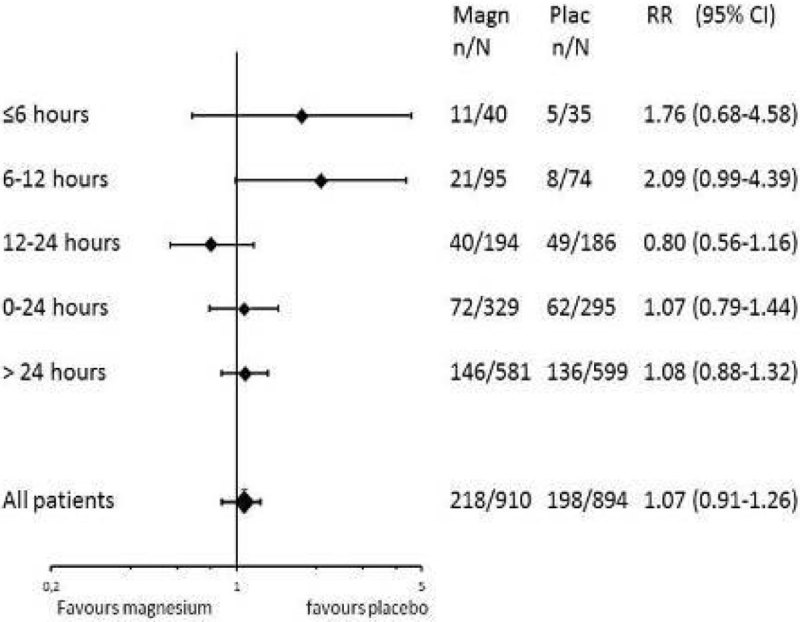
**Adjusted RR for occurrence of DCI for magnesium ve.**

**Figure 2:**
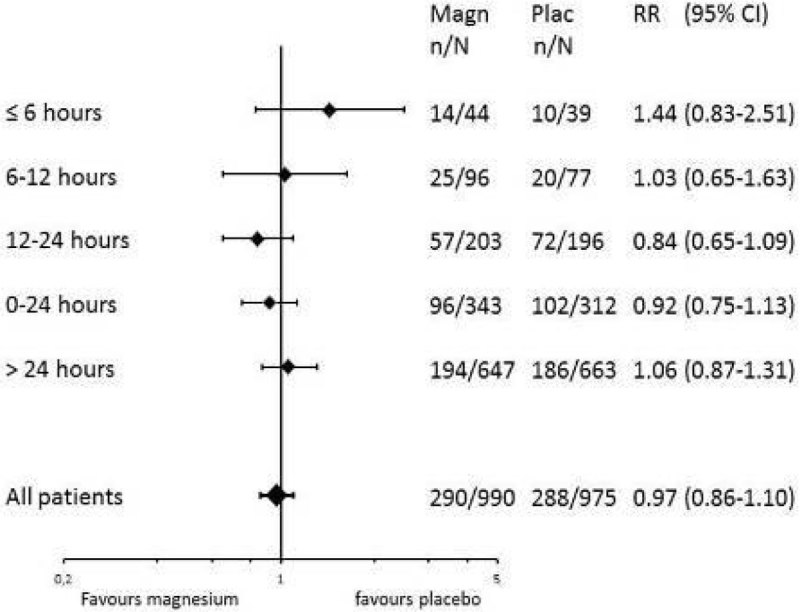
**Adjusted RR for poor outcome for magnesium versus.**

